# Association of the Time to First Cigarette and the Prevalence of Chronic Respiratory Diseases in Chinese Elderly Population

**DOI:** 10.2188/jea.JE20200502

**Published:** 2022-09-05

**Authors:** Chao Wang, Heng Jiang, Yi Zhu, Yingying Guo, Yong Gan, Qingfeng Tian, Yiling Lou, Shiyi Cao, Zuxun Lu

**Affiliations:** 1 Department of Social Medicine and Health Management, School of Public Health, Tongji Medical College, Huazhong University of Science and Technology, Wuhan, Hubei, China; 2 Centre for Alcohol Policy Research, School of Psychology and Public Health, La Trobe University, Melbourne, VIC, Australia; 3 Centre for Health Equity, Melbourne School of Population and Global Health, University of Melbourne, Melbourne, VIC, Australia; 4 Tongji Hospital, Tongji Medical College, Huazhong University of Science and Technology, Wuhan, Hubei, China; 5 Department of Social Medicine and Health Management, School of Public Health, Zhengzhou University, Zhengzhou, Henan, China

**Keywords:** time to first morning cigarette, chronic respiratory diseases, Chinese elderly, Chinese Longitudinal Healthy Longevity Survey

## Abstract

**Background:**

Increasing number of studies have suggested the time to first cigarette after waking (TTFC) have significant positive effect on respiratory diseases. However, few of them focused on the Chinese population. This study aims to estimate the impact of TTFC on the prevalence of chronic respiratory diseases (CRD) in Chinese elderly and explore the association in different sub-populations.

**Methods:**

Cross-sectional data of demographic characteristics, living environment, smoking-related variables, and CRD were drawn from the Chinese Longitudinal Healthy Longevity Survey in 2018. Multivariate stepwise logistic regression analyses were conducted to examine the association of the TTFC with the prevalence of CRD.

**Results:**

This study includes 13,208 subjects aged 52 years and older, with a mean age of 85.3 years. Of them, 3,779 participants were ex- or current smokers (44.9% had the TTFC ≤30 minutes, 55.1% >30 minutes) and 1,492 had suffered from CRD. Compared with non-smokers, participants with TTFC ≤30 minutes seemed to have higher prevalence of CRD (OR 1.97; 95% CI, 1.65–2.35) than those with TTFC >30 minutes (OR 1.70; 95% CI, 1.44–2.00), although the difference was statistically insignificant (*P_interaction_* = 0.12). Compared with TTFC >30 minutes, TTFC ≤30 minutes could drive a higher prevalence of CRD among female participants, those aged 90 years and older, urban residents, and ex-smokers (*P_interaction_* < 0.05).

**Conclusion:**

Shorter TTFC relates to higher prevalences of CRD in Chinese older females, those aged 90 years and older, urban residents, and ex-smokers. Delaying TTFC might partially reduce its detrimental impact on respiratory disease in these specific subpopulations.

## INTRODUCTION

Respiratory disease is an umbrella term that includes bronchitis, emphysema, asthma, and pneumonia, and it is associated with significant morbidity and mortality among the older population.^1^^,^^2^ Smoking has been determined as one of the leading risk factors of respiratory disease.^3^^–^^5^ In addition to the traditional predictor valuables of smoking intensity and consumption duration, the time to first cigarette after waking (TTFC) has been suggested as a novel proxy of nicotine dependence and could be more valuable in precisely assessing smokers’ risk of respiratory disease.^6^^,^^7^ For example, people with a shorter TTFC have a higher risk of asthma,^8^ chronic obstructive pulmonary disease,^9^ and lung cancer.^10^

There are great disparities in smoking pattern among smokers with various sociocultural backgrounds and genetic phenotypes, which might be responsible for ethnic disparities in smoking-related health consequences.^11^^,^^12^ While a number of existing studies only focused on the relationship between the TTFC and the risk of respiratory disease among specific population, rather than comprehensive subgroup analyses across sub-populations of smokers (such as those stratified by gender, age, smoking status), so it has limited the application of study findings to some extent. Considering that smokers today are increasingly likely to have their first cigarette earlier on awakening than they were a decade ago,^13^ national studies are warranted to explore heterogeneity within ethnic groups or sub-populations.

With the population aging worldwide, global burden of late-life disease has steadily increased and smoking-related diseases were one of outstanding challenges of them.^14^^,^^15^ About 167 million Chinese, accounting for 11.9% of the total population, were 65 years and older at the end of 2018, and the aging is accelerating.^16^ Furthermore, with lung function decline, such as loss of lung elasticity and reduced thoracic cage movement,^17^ the prevalence of respiratory diseases are high among the elderly comparing to the youth.^18^ The TTFC has been identified as a valid indicator for the risk of respiratory disease in Europeans and Americans; however, no study has been conducted to examine the relationship between the TTFC and the risk of respiratory disease of China’s elderly population yet. With the data of the Chinese Longitudinal Healthy Longevity Survey (CLHLS), this study aims to investigate the association between the TTFC and the prevalence of chronic respiratory diseases (CRD) among the Chinese elder people and further explore the association in different sub-populations.

## METHODS

### Study design and participants

**Figure 1.**
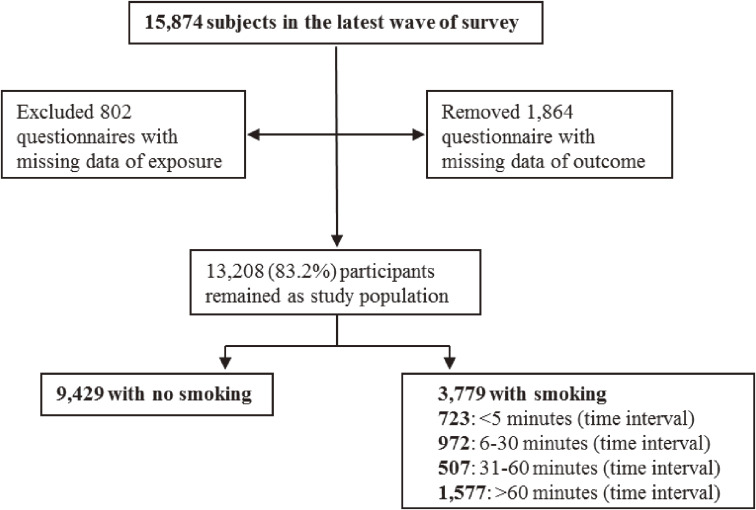
The flow chart of the screen of eligible data

The CLHLS is an ongoing, prospective, and national study of community-dwelling Chinese older people started from 1998, and follow-up interview was performed every 2 to 3 years. The rationale, design and methods of this survey have been previously described in detail.^19^^–^^21^ Briefly, this survey includes about 16,000 elderly people from more than 500 sample points in 22 province-level administrative regions across China. The surveys are administered in participants’ homes by trained interviewers with a structured questionnaire. Proxy respondents, usually a spouse or other close family members, are interviewed when participants are unable to answer questions. In this study, we interviewed all participants (*n* = 15,874) enrolled in the latest wave of the survey in 2018. After excluding 802 questionnaires with missing data of exposure (the TTFC) and 1,864 with missing data of CRD (suffering from bronchitis/emphysema/asthma/pneumonia or not), 13,208 respondents were ultimately included as study population, yielding a qualification rate of 83.2% (Figure 1).

The CLHLS was approved by the Research Ethics Committee of Peking University (IRB00001052-13074), and all participants or their proxy respondents provided written informed consent.

### Data collection

The TTFC was measured based on smoker’s answer to the question of “How soon do you smoke your first cigarette after you wake up?”. Then, we dichotomized the TTFC into two groups: ≤30 minutes and >30 minutes. Smoking status were recorded and divided into three categories (never, ex-, and current) according to the questionnaire. The outcome (the prevalence of respiratory disease) in this study was defined based on a yes or no answer to the question of “Are you suffering from bronchitis/emphysema/asthma/pneumonia that has been diagnosed?”

We further collected information of participants from following four domains: 1) demographic characteristics (eg, sex, age, rurality, years of schooling, marital status, body mass index [BMI; underweight: BMI <18.5 kg/m^2^; normal weight: BMI 18.5–23.9 kg/m^2^; overweight/obesity: BMI ≥24.0 kg/m^2^]^22^); 2) lifestyle factors (eg, alcohol consumption, physical exercise [referring to purposeful fitness activities, such as walking, playing ball, running, and qigong], sleep time); 3) household environment (eg, walking time to the nearest medical institution, distance to the arterial road, fuel of cooking, ventilation of kitchen, air cleaning in household, living expenses); 4) smoking related factors (eg, past-present smoking status, smoking times per day, years of smoking, smoking amount per day of housemates). For the covariates with missing values of no more than 5%, we used the multiple imputation to replace the missing data with one or more specific values, results were combined using the standard rules from Rubin.^23^ Finally, only a small percentage of the data for most covariates were missing (<2%), with the exception of fuel for cooking (3.8%) and ventilation of the kitchen (4.5%).

### Statistical analysis

Data of the survey were inputted into the SPSS software (Version 22.0, SPSS Inc, Chicago, IL, USA) for statistical analyses. Continuous variables were described as mean (standard deviation [SD]), and frequency data were expressed as number (%). We analyzed the demographic statistics using the *χ*^2^ test. Forward stepwise multivariate logistic regression analysis (level for selection: *P* ≤ 0.05, level for elimination: *P* ≥ 0.10) were used to estimate the association between the TTFC and prevalence of CRD among the Chinese elder people, with non-smokers as the reference group. We also tested the statistical significance of the difference of the estimates.^24^

We did several sensitivity analyses (models 2–7), where we added different covariates into model 1, to examine the robustness of the primary results. In model 2, we controlled only for age and sex. In model 3–5, we added covariates mentioned above into models step-by-step. In model 6, we controlled only past-present smoking status and smoking amount per day. In model 7, we included all covariates. Furthermore, we also did subgroup analyses stratified by sex, age, rurality, past-present smoking status and smoking amount per day, respectively, to study the impact of TTFC on CRD in different sub-populations. The significance level was accepted as *P* < 0.05 (two-sided) for all tests.

## RESULTS

We included 13,208 participants in this study. The mean age was 85.3 (SD, 11.7) years old, and 7,456 (56.5%) participants were female. Over a quarter (28.6%) of participants reported smoking. Of them, 1,747 respondents (46.2%) were ex-smokers, and 2,013 respondents (53.8%) were current smokers; 1,695 respondents (44.9%) had the TTFC ≤30 minutes, 2,084 respondents (55.1%) reported the TTFC >30 minutes; 2,027 respondents (53.6%) smoked ≤10 times per day, and 1,752 respondents (46.4%) smoked >10 times per day. A total of 1,492 (11.3%) respondents have suffered from the CRD (Table 1).

**Table 1.** The univariate analysis of the prevalence of chronic respiratory diseases among people with different demographic characteristics (*n* = 13,208)

Variables	*N* (%)	Unsuffered (%)	Suffered (%)	*χ* ^2^	*P*
**Sex**					
Male	5,752 (43.5)	4,944 (86.0)	808 (14.0)	76.96	<0.001
Female	7,456 (56.5)	6,772 (90.8)	684 (9.2)		
**Age, years**					
52–79	4,597 (34.8)	4,165 (90.6)	432 (9.4)	40.20	<0.001
80–89	3,330 (25.2)	2,865 (86.0)	465 (14.0)		
≥90	5,281 (40.0)	4,686 (88.7)	595 (11.3)		
**Rurality** ^a^					
Urban residents	3,607 (27.4)	3,099 (85.9)	508 (14.1)	40.43	<0.001
Rural residents	9,548 (72.6)	8,578 (89.8)	970 (10.2)		
**Years of schooling**					
No schooling	5,564 (42.1)	4,976 (89.4)	588 (10.6)	5.09	0.078
1–6 years of schooling	4,883 (37.0)	4,305 (88.2)	578 (11.8)		
≥7 years of schooling	2,761 (20.9)	2,435 (88.2)	326 (11.8)		
**Marital status** ^b^					
Married and live together	5,187 (39.6)	4,586 (88.4)	601 (11.6)	0.88	0.349
Never married/separated/divorced/widowed	7,895 (60.4)	7,022 (88.9)	873 (11.1)		
**BMI, kg/m^2^**					
Underweight (<18.5)	6,804 (51.5)	6,030 (88.6)	774 (11.4)	2.93	0.232
Normal (18.5–23.9)	2,262 (17.1)	1,988 (87.9)	274 (12.1)		
Overweight/Obese (≥24)	4,142 (31.4)	3,698 (89.3)	444 (10.7)		
**Physical exercise** ^c^					
Yes	3,917 (30.0)	3,506 (89.5)	411 (10.5)	3.36	0.067
No physical exercise	9,129 (70.0)	8,070 (88.4)	1,059 (11.6)		
**Alcohol consumption** ^d^					
Yes	1,874 (14.4)	1,692 (90.3)	182 (9.7)	5.60	0.018
No	11,162 (85.6)	9,869 (88.4)	1,293 (11.6)		
**Sleep time**					
<7 hours	4,753 (36.0)	4,145 (87.2)	608 (12.8)	18.35	<0.001
7–9 hours	5,551 (42.0)	4,989 (89.9)	562 (10.1)		
≥10 hours	2,904 (22.0)	2,582 (88.9)	322 (11.1)		
**Physical examination** ^e^					
Regular physical examination	8,910 (67.8)	7,912 (88.8)	998 (11.2)	0.27	0.601
No physical examination	4,240 (32.2)	3,752 (88.5)	488 (11.5)		
**Fuel for cooking** ^f^					
No cooking	225 (1.8)	199 (88.4)	26 (11.6)	7.41	0.025
Electricity/Gas	8,654 (68.1)	7,638 (88.3)	1,016 (11.7)		
Coal/Firewood/Straw	3,822 (30.1)	3,437 (89.9)	385 (10.1)		
**Ventilation of the kitchen** ^g^					
No ventilation	1,140 (9.0)	1,027 (90.1)	113 (9.9)	9.68	0.021
Lampblack exhauster	4,364 (34.6)	3,826 (87.7)	538 (12.3)		
Ventilation hood	950 (7.5)	844 (88.8)	106 (11.2)		
Nature windowing ventilation	6,166 (48.9)	5,512 (89.4)	654 (10.6)		
**Air cleaning in household** ^h^					
Yes	1,042 (8.0)	910 (87.3)	132 (12.7)	2.42	0.12
No	11,956 (92.0)	10,631 (88.9)	1,325 (11.1)		
**Distance to the arterial road** ^i^					
<100 m	4,081 (31.3)	3,563 (87.3)	518 (12.7)	14.81	0.002
101–300 m	2,350 (18.1)	2,083 (88.6)	267 (11.4)		
>300 m	5,732 (44.0)	5,138 (89.6)	594 (10.4)		
Not sure	855 (6.6)	771 (90.2)	84 (9.8)		
**Distance to the nearest hospital** ^j^					
<1 km	1,358 (10.4)	1,170 (86.2)	188 (13.8)	11.47	0.009
1.0–2.9 km	8,094 (61.8)	7,216 (89.2)	878 (10.8)		
3.0–4.9 km	1,783 (13.6)	1,591 (89.2)	192 (10.8)		
≥5 km	1,858 (14.2)	1,638 (88.2)	220 (11.8)		
**Daily smoking per day of persons living together** ^k^					
No cigarettes	6,374 (61.3)	5,660 (88.8)	714 (11.2)	14.77	0.002
1–10 cigarettes	1,442 (13.9)	1,282 (88.9)	160 (11.1)		
11–20 cigarettes	1,455 (14.0)	1,310 (90.0)	145 (10.0)		
>20 cigarettes	1,123 (10.8)	959 (85.4)	164 (14.6)		
**Years of smoking**					
No smoking	9,429 (71.4)	8,543 (90.6)	886 (9.4)	127.11	<0.001
<30 years	682 (5.2)	588 (86.2)	94 (13.8)		
30–49 years	1,203 (9.1)	999 (83.0)	204 (17.0)		
50–69 years	1,403 (10.6)	1,163 (82.9)	240 (17.1)		
≥70 years	491 (3.7)	423 (86.2)	68 (13.8)		
**TTFC**					
No smoking	9,429 (71.4)	8,543 (72.9)	886 (9.4)	121.48	<0.001
TTFC ≤30 minutes	1,695 (12.8)	1,407 (83.0)	288 (17.0)		
TTFC >30 minutes	2,084 (15.8)	1,766 (84.7)	318 (15.3)		
**Present-past smoking status** ^l^					
Non-smoker	9,429 (71.5)	8,543 (90.6)	886 (9.4)	170.12	<0.001
Ex-smoker	1,747 (13.2)	1,397 (80.0)	350 (20.0)		
Current smoker	2,013 (15.3)	1,762 (87.5)	251 (12.5)		
**Daily cigarette consumption**					
No smoking	9,429 (71.4)	8,543 (90.6)	886 (9.4)	126.46	<0.001
1–10 cigarette	2,027 (15.3)	1,729 (85.3)	298 (14.7)		
>10 cigarette	1,752 (13.3)	1,444 (82.4)	308 (17.6)		

BMI, body mass index; TTFC, time to first morning cigarette after waking.

^a^53 data were missing.

^b^126 data were missing.

^c^162 data were missing.

^d^172 data were missing.

^e^58 data were missing.

^f^507 data were missing.

^g^588 data were missing.

^h^210 data were missing.

^i^190 data were missing.

^j^115 data were missing.

^k^31 data were missing.

^l^19 data were missing.

Univariate analysis (Table 1) shows that sex, age, rurality, alcohol consumption, sleep time, ventilation of kitchen, distance to the nearest medical institution, fuels of cooking, distance to the arterial road, past-present smoking status, smoking times per day, years of smoking, and smoking amount of housemates were all significantly associated with the prevalence of CRD (*P* < 0.05).

**Figure 2.**
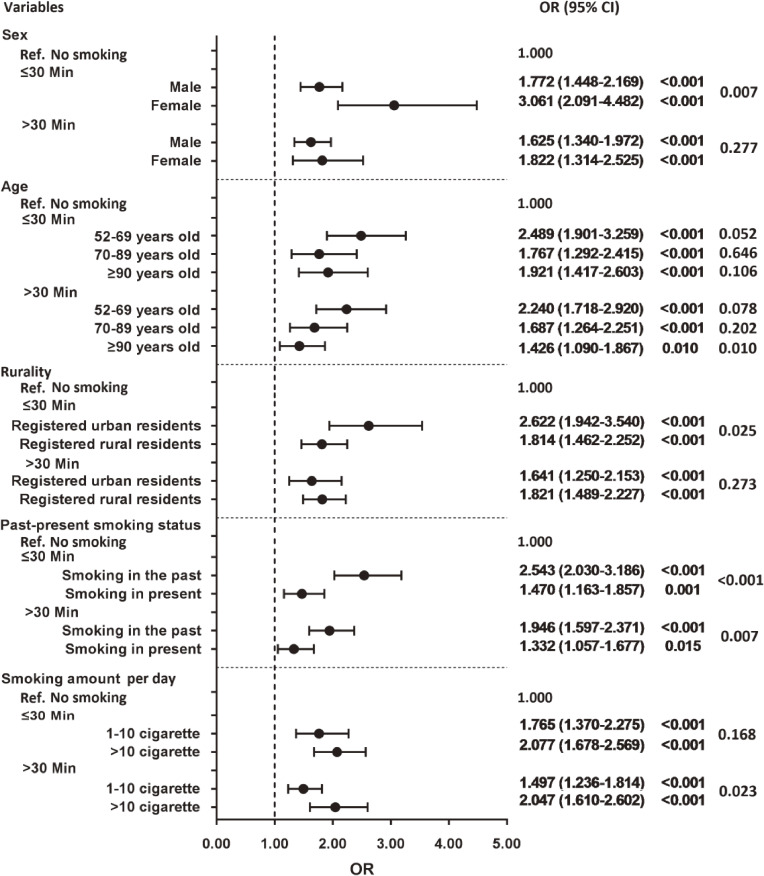
Subgroup analyses on the relationship between the TTFC and the prevalence of CRD stratified by sex, age, rurality, past-present smoking status, and smoking amount per day. CI, confidence interval; CRD, chronic respiratory diseases; OR, odds ratio; Ref., reference; TTFC, time to first morning cigarette after waking.

Table 2 shows that the association between the TTFC and the prevalence of CRD through forward stepwise multivariable logistic regression analyses. Compared with non-smokers, respondents with the TTFC ≤30 minutes were 1.97 times more likely to report prevalence of CRD (OR 1.97; 95% CI, 1.65–2.35), while respondents with TTFC >30 minutes were 1.70 times more (OR 1.70; 95% CI, 1.44–2.00). However, the difference between two estimates was statistically insignificant (*P_interaction_* = 0.12). The association of TTFC and the prevalence of CRD remained significant after adjusting for demographic characteristics and other covariates (eTable 1, eTable 2, eTable 3, eTable 4, eTable 5, and eTable 6). In subgroup analyses, the effect of the TTFC on the prevalence of CRD was significantly different by sex, age, rurality, past-present smoking status, or smoking amount per day. In the female group, compared with those had the TTFC >30 minutes, respondents with the TTFC ≤30 minutes shown significantly higher prevalence of CRD (3.06 vs 1.82). Similar results were also found among respondents aged 90 years and older (1.92 vs 1.43), urban residents (2.62 vs 1.64), and those ex-smokers (2.54 vs 1.95) (all *P_interaction_* < 0.05). (Figure 2 and eTable 8, eTable 9, eTable 10, eTable 11, and eTable 12).

**Table 2.** Stepwise multivariate logistic regression analyses on the associations between TTFC and the prevalence of chronic respiratory diseases in seven models

Models	Adjusted covariant	TTFC, minutes			*P_interaction_*

		No smoking (Ref.)	≤30 OR (95% CI)	>30 OR (95% CI)	
**Model 1**	None	1.000	1.97 (1.71–2.28)	1.74 (1.51–1.99)	0.104
**Model 2**	Sex, Age	1.000	1.76 (1.49–2.07)	1.54 (1.32–1.80)	0.123
**Model 3**	Sex, Age, Rurality, Years of schooling, Marital status, BMI	1.000	1.83 (1.55–2.16)	1.58 (1.36–1.85)	0.107
**Model 4**	Sex, Age, Rurality, Alcohol consumption, Physical exercise, BMI, Sleep time, Years of schooling, Marital status	1.000	1.99 (1.68–2.36)	1.71 (1.46–2.01)	0.101
**Model 5**	Sex, Age, Rurality, Ventilation in kitchen, Distance to the nearest medical institution, Physical examination, Alcohol consumption, Physical exercise, air cleaning in household, Lives expense, BMI, Sleep time, Years of schooling, Marital status, Fuel of cooking, Distance to the arterial traffic	1.000	1.98 (1.66–2.36)	1.69 (1.43–2.00)	0.103
**Model 6**	Daily smoking per day of persons living together, Years of smoking	1.000	1.97 (1.70–2.27)	1.74 (1.52–2.00)	0.117
**Model 7**	All adjusted	1.000	1.97 (1.65–2.35)	1.70 (1.44–2.00)	0.118

BMI, body mass index; CI, confidence interval; OR, odds ratio; Ref., reference; TTFC, time to first morning cigarette after waking.

## DISCUSSION

This study has presented a comprehensive analysis on the association between the TTFC and the prevalence of CRD among older Chinese population using a large scale national representative sample. We found both the shorter (≤30 minutes) and longer (>30 minutes) TTFC were positively associated with the prevalence of CRD among the Chinese elderly, but there was no statistically significant difference between the two estimates. This finding was inconsistent with that of previous studies conducted in other countries.^6^^,^^9^^,^^10^^,^^25^ However, compared with TTFC >30 minutes, TTFC ≤30 minutes could significantly increase the prevalence of CRD among females, individuals aged 90 years and older, urban residents, and ex-smokers, suggesting that these sub-groups should be priority populations of health screening and tobacco control related to TTFC.

Smoking is one of the important contributions for the chronic airway inflammation,^26^ which enhances excitability of vagal bronchopulmonary sensory nerves and increase tachykinin synthesis, causing the airway hyper-responsiveness and consequent respiratory disease.^27^ People breathe slowly and smoothly during sleep,^28^ on waking up, the breath volume and frequency increase immediately. In this case, morning cigarette smoking could heighten its toxic stimulation on respiratory system. For example, asthma is worse in the morning as compared to the evening.^29^ Nicotine dependence could be another plausible mechanism. A population-based study has shown that the highly dependent smokers inhale cigarette smoke more deeply and retain it in airways longer.^30^ Thus a higher dose of harmful cigarette smoke components enter body and a longer toxicity exposure aggravates the inflammation of respiratory system.

In contrast to American^31^ and European^10^ studies, we found no significant difference in associations between shorter (≤30 minutes) and longer (>30 minutes) TTFC with the prevalence of CRD among older smokers in China. This disparity could be explained by the lower level of nicotine dependence in Chinese than that in other racial groups.^32^^,^^33^ First, TTFC is arguably the best single-item measure of nicotine dependence^6^ and, on average, Asians tend to have longer TTFC than the Europeans and Americans,^32^^,^^34^ which is partly evidenced in our study, showing that a larger proportion of TTFC with over 60 minutes in Chinese elderly compared with the European/American population (15.3% vs 13.2%). Second, nicotine dependence and smoking consumption are maintained by the pharmacologic effect of nicotine. Among Asians, a lower enzymatic activity of the CYP2A6^*^4 was detected,^35^ which can slow down the metabolism of the nicotine and cotinine (the primary proximate metabolite of nicotine).^36^^,^^37^ In addition, a study examining the nicotine toxicity following patch placement also found that the probability of nicotine-induced toxicity tended to be lower among Asians comparing with white people.^38^ Based on this evidence, we hypothesized that due to the relatively lower nicotine dependence and slower nicotine toxicity accumulation, the detrimental impact of tobacco on the respiratory system of the elderly Chinese smokers could be delayed to some extent. As the different distribution of TTFC among different ethnic groups, the threshold for TTFC predicting the prevalence of CRD should be altered. A higher cut-off point of TTFC might be more suitable for exploring the relationship with the occurrence of CRD in older Chinese smokers.

Women appear to be more vulnerable to cigarette smoking^39^ and maintain a higher prevalence of respiratory disease.^40^ Throughout the human life span, female lungs tend to be smaller and to weigh less than male lungs, correspondingly, their airways are narrower in diameter than that of men.^41^ However, they exhibit higher forced expiratory flow rates, and the ratio of forced expiratory volume in one second to forced vital capacity ratios are higher in women.^42^ When smoking, women’s respiratory tracts are under more pressure, accelerating the toxic impact of tobacco composition on the tracheal cells. Except the difference in physiological structure, different nicotine metabolism between sexes could be another possible reason of the gender difference. Women generally metabolize nicotine more rapidly than men^43^^,^^44^; thus, when consuming the same amount of nicotine/cigarettes, women may have higher exposure to harmful substances of cigarettes than men.

Residents living in urban areas expose to more aerial pollution, which has been identified to be interacted with cigarette smoking, than those in rural areas, the combined effect is more detrimental to people’s respiratory health.^45^ In addition, the higher diagnosis rate of CRD among urban residents could partially attribute to their more convient access to high-quality medical or healthcare services. In the past, false tobacco advertisement, poorer design (eg, no filter tip), and adverse cigarette products (containing more tar, nicotine, or carbon monoxide) generated more harms to nowdays older population than those younger.^46^^,^^47^ Plus China’s traditional smoking beliefs and culture (cigarette gifting and sharing),^48^ the hazardous impact of smoking on the elderly smokers could be larger. Furthermore, our study also found that the shorter TTFC was more closely associated with the presence of chronic respiratory diseases than the longer TTFC in ex-smoker, while not in current smoker. There was nonetheless evidence of survivor bias in our study for the inevitable questions of the cross-section design. The current smokers in our analysis are generally healthier than those ex-smokers, as the latter might need to quit smoking because of smoke-related diseases. Moreover, a combination of survival bias, so-called healthy-smoker bias, and recall bias about the details of remote smoking history all could lead to these results.

This is the first national study on the association of the TTFC and the prevalence of CRD in Chinese elderly population. Most previous studies on this topic were conducted in other countries or focused on younger adults.^49^^,^^50^ In fact, smoking control in older people could bring significant gains in healthy life expectancy^51^ and lower risks of disability.^52^ Therefore, this study has clinical and public health implications, and the findings provide a novel insight for smoking-related health education. Further, participants in this study were from the nationwide survey of CLHLS, which covers approximate 85% of China’s citizens,^21^ so the findings have appropriate representativeness and applicability.

Some limitations should be noted. First, this is only one round of the CLHLS survey, the data of this study limited its ability to identify the causal relationships between the TTFC and the prevalence of CRD. Second, we adopt composite endpoints, the CRD, in this study, which has made it difficult to assess the impact of the TTFC on the risk of specific endpoint event. To clarify the findings, further studies with separate respiratory disease as outcome should be conducted. Third, smoking exposure (from conventional cigarettes or from e-cigarettes and heated tobacco products) was not specified in this study, application of the findings to those who use e-cigarettes and heated tobacco products should be careful. Fourth, some comparative groups involved in the discussion were Asians or Chinese origin population, rather than residents living in China, it could limit the findings implication to some extent considering disparities of lifestyle and environmental exposures.

In conclusion, the difference between shorter (≤30 minutes) and longer (>30 minutes) TTFC on the prevalence of CRD was statistically insignificant in Chinese elderly. However, the shorter TTFC might increase the prevalence of CRD in females, people aged 90 years and older, urban residents, and ex-smokers. Our findings suggest that the use of TTFC as an indicator of the prevalence of CRD in elderly Chinese smokers may not be as applicable as in the European and American populations, but the underlying relationship of TTFC and the prevalence of CRD among specific Chinese population warrants further research.

## SUPPLEMENTARY MATERIAL

eTable 1. Relationship of the TTFC and the risk of chronic respiratory diseases, model 1 (unadjusted model)

eTable 2. Relationship of the TTFC and the risk of chronic respiratory diseases, model 2 (adjusted for sex, age)

eTable 3. Relationship of the TTFC and the risk of chronic respiratory disease, model 3 (adjusted for sex, age, rurality, alcohol consumption, physical exercise, BMI)

eTable 4. Relationship of the TTFC and the risk of chronic respiratory diseases, model 4 (adjusted for sex, age, hukou, alcohol consumption, physical exercise, BMI, sleep time, years of schooling, marital status)

eTable 5. Relationship of the TTFC and the risk of chronic respiratory diseases, model 5 (adjusted for sex, age, hukou, ventilation in kitchen, distance to the nearest medical institution, physical examination, alcohol consumption, physical exercise, air cleaning in household, lives expense, BMI, sleep time, years of schooling, marital status, fuel of cooking, distance to the arterial traffic)

eTable 6. Relationship of the TTFC and the risk of chronic respiratory diseases, model 6 (adjusted for smoking status of people living together, years of smoking)

eTable 7. Relationship of the TTFC and the risk of chronic respiratory diseases, model 7 (fully adjusted)

eTable 8. Subgroup analyses on the relationship between the time interval between the first cigarette smoking in the morning and waking up and the risk of chronic respiratory diseases stratified by sex

eTable 9. Subgroup analyses on the relationship between the time interval between the first cigarette smoking in the morning and waking up and the risk of chronic respiratory diseases stratified by age

eTable 10. Subgroup analyses on the relationship between the time interval between the first cigarette smoking in the morning and waking up and the risk of chronic respiratory diseases stratified by rurality

eTable 11. Subgroup analyses on the relationship between the time interval between the first cigarette smoking in the morning and waking up and the risk of chronic respiratory diseases stratified by smoking status

eTable 12. Subgroup analyses on the relationship between the time interval between the first cigarette smoking in the morning and waking up and the risk of chronic respiratory diseases stratified by smoking consumption
