# Supplementary material for: Association of the Time to First Cigarette and the Prevalence of Chronic Respiratory Diseases in Chinese Elderly Population
Source: J Epidemiol. 2022 Sep 5;32(9):415–22. doi: 10.2188/jea.JE20200502 (PMC9359902; doi:10.2188/jea.JE20200502)
Supplement: Supplementary file 1 [file je-32-415-s001.pdf]

eTable 1. Relationship of the TTFC and the risk of chronic respiratory diseases, model 1 (unadjusted model)

	**B**	**S.E.**	**Wald**	**P**	**OR**	**95% CI**	
						**LL**	**UL**
**TTFC (Ref. No smoking)**							
≤30 minutes	0.68	0.07	85.15	0.000	1.97	1.71	2.28
>30 minutes	0.55	0.07	61.41	0.000	1.74	1.51	1.99
**Constant**	-2.27	0.04	4122.46	0.000	0.10		

CI, confidence interval; LL, lower limit; OR, odds ratio; Ref., reference; S.E., standard error; TTFC, time to first morning cigarette after waking; UL, upper limit.

eTable 2. Relationship of the TTFC and the risk of chronic respiratory diseases, model 2 (adjusted for sex, age)

	**B**	**S.E.**	**Wald**	**P**	**OR**	**95% CI**	
						**LL**	**UL**
**TTFC (Ref. No smoking)**							
≤30 minutes	0.57	0.08	45.88	0.000	1.76	1.49	2.07
>30 minutes	0.43	0.08	30.17	0.000	1.54	1.32	1.80
**Sex (Ref. Male)**	-0.26	0.07	15.48	0.000	0.77	0.68	0.88
**Age, years (Ref. 52–69 years old)**							
70–89	0.49	0.07	45.89	0.000	1.63	1.41	1.87
≥90	0.32	0.07	22.63	0.000	1.38	1.21	1.58
**Constant**	-2.36	0.07	1091.58	0.000	0.10		

CI, confidence interval; LL, lower limit; OR, odds ratio; Ref., reference; S.E., standard error; TTFC, time to first morning cigarette after waking; UL, upper limit.

eTable 3. Relationship of the TTFC and the risk of chronic respiratory diseases, model 3 (adjusted for sex, age, rurality, alcohol consumption, physical exercise, BMI)

	B	S.E.	Wald	P	OR	95% CI	
						LL	UL
**TTFC (Ref. No smoking)**							
≤30 minutes	0.60	0.09	50.74	0.000	1.83	1.55	2.16
>30 minutes	0.46	0.08	33.46	0.000	1.58	1.36	1.85
**Sex (Ref. Male)**	-0.22	0.07	10.56	0.001	0.81	0.71	0.92
**Age, years (Ref. 52–69 years old)**							
70–89	0.49	0.07	44.82	0.000	1.62	1.41	1.87
≥90	0.33	0.07	22.28	0.000	1.38	1.21	1.58
**Rurality (Ref. Registered urban residents)**	-0.39	0.06	43.14	0.000	0.68	0.60	0.76
**Constant**	-2.13	0.08	701.48	0.000	0.12		

CI, confidence interval; LL, lower limit; OR, odds ratio; Ref., reference; S.E., standard error; TTFC, time to first morning cigarette after waking; UL, upper limit.

eTable 4. Relationship of the TTFC and the risk of chronic respiratory diseases, model 4 (adjusted for sex, age, hukou, alcohol consumption, physical exercise, BMI, sleep time, years of schooling, marital status)

	B	S.E.	Wald	P	OR	95% CI	
						LL	UL
**TTFC (Ref. No smoking)**							
≤30 minutes	0.69	0.09	62.89	0.000	1.99	1.68	2.36
>30 minutes	0.54	0.08	43.80	0.000	1.71	1.46	2.01
**Sex (Ref. Male)**	-0.32	0.07	21.34	0.000	0.73	0.64	0.83
**Age, years (Ref. 52–69 years old)**							
70–89	0.44	0.07	35.76	0.000	1.56	1.35	1.80
≥90	0.27	0.07	13.57	0.000	1.31	1.13	1.51
**Rurality (Ref. Urban residents)**	-0.43	0.06	48.22	0.000	0.65	0.58	0.74
**Alcohol consumption (Ref. Consumption)**	0.48	0.09	28.29	0.000	1.61	1.35	1.92
**Physical exercise (Ref. Having exercise)**	0.24	0.07	13.23	0.000	1.27	1.12	1.45
**Sleep time (Ref. <7 hours)**							
7–9 hours	-0.27	0.06	18.11	0.000	0.76	0.67	0.86
≥10 hours	-0.25	0.08	9.99	0.002	0.78	0.67	0.91

CI, confidence interval; LL, lower limit; OR, odds ratio; Ref., reference; S.E., standard error; TTFC, time to first morning cigarette after waking; UL, upper limit.

eTable 5. Relationship of the TTFC and the risk of chronic respiratory diseases, model 5 (adjusted for sex, age, hukou, ventilation in kitchen, distance to the nearest medical institution, physical examination, alcohol consumption, physical exercise, air cleaning in household, lives expense, BMI, sleep time, years of schooling, marital status, fuel of cooking, distance to the arterial traffic)

	B	S.E.	Wald	P	OR	95% CI	
						LL	UL
**TTFC (Ref. No smoking)**							
≤30 minutes	0.69	0.09	57.72	0.000	1.99	1.66	2.37
>30 minutes	0.54	0.08	40.67	0.000	1.71	1.45	2.02
**Sex (Ref. Male)**	-0.31	0.07	19.30	0.000	0.73	0.64	0.84
**Age, years (Ref. 52–69 years old)**							
70–89	0.42	0.08	30.66	0.000	1.53	1.31	1.77
≥90	0.26	0.08	11.56	0.001	1.29	1.12	1.50
**Rurality (Ref. Urban residents)**	-0.44	0.07	43.80	0.000	0.64	0.56	0.73
**Distance to medical institutions (Ref. <1.0 km)**							
1.0–2.9 km	-0.22	0.09	5.38	0.02	0.80	0.67	0.97
3.0–4.9 km	-0.20	0.12	2.85	0.091	0.82	0.65	1.03
≥5.0 km	-0.01	0.12	0.00	0.964	1.00	0.79	1.25
**Alcohol consumption (Ref. Drinking)**	0.48	0.09	26.58	0.000	1.61	1.34	1.93
**Physical exercise (Ref. Having exercise)**	0.21	0.07	9.11	0.003	1.23	1.08	1.41
**Living expense (Ref. Sufficient)**	0.18	0.08	4.81	0.028	1.20	1.02	1.41
**Sleep time (Ref. <7 hours)**							
7–9 hours	-0.26	0.07	14.91	0.000	0.77	0.68	0.88
≥10 hours	-0.24	0.08	8.30	0.004	0.79	0.67	0.93
**Constant**	-2.29	0.15	246.05	0.000	0.10		

CI, confidence interval; LL, lower limit; OR, odds ratio; Ref., reference; S.E., standard error; TTFC, time to first morning cigarette after waking; UL, upper limit.

eTable 6. Relationship of the TTFC and the risk of chronic respiratory diseases, model 6 (adjusted for smoking status of people living together, years of smoking)

	B	S.E.	Wald	P	OR	95% CI	
						LL	UL
**TTFC (Ref. No smoking)**							
≤30 minutes	0.68	0.07	83.74	0.000	1.97	1.70	2.27
>30 minutes	0.55	0.07	61.96	0.000	1.74	1.52	2.00

CI, confidence interval; LL, lower limit; OR, odds ratio; Ref., reference; S.E., standard error; TTFC, time to first morning cigarette after waking; UL, upper limit.

eTable 7. Relationship of the TTFC and the risk of chronic respiratory diseases, model 7 (fully adjusted)

	B	S.E.	Wald	P	OR	95% CI	
						LL	UL
**TTFC (Ref. No smoking)**							
≤30 minutes	0.68	0.09	56.42	0.000	1.98	1.65	2.36
>30 minutes	0.54	0.08	41.17	0.000	1.72	1.46	2.02
**Sex (Ref. Male)**	-0.31	0.07	19.16	0.000	0.73	0.64	0.84
**Age, years (Ref. 52–69 years old)**							
70–89	0.42	0.08	30.32	0.000	1.52	1.31	1.77
≥90	0.25	0.08	10.86	0.001	1.28	1.11	1.49
**Rurality (Ref. Urban residents)**	-0.45	0.07	44.28	0.000	0.64	0.56	0.73
**Distance to medical institutions (Ref. <1.0 km)**							
1.0–2.9 km	-0.22	0.09	5.52	0.019	0.80	0.67	0.96
3.0–4.9 km	-0.22	0.12	3.17	0.075	0.81	0.64	1.02
≥5.0 km	-0.00	0.12	0.00	0.990	1.00	0.80	1.25
**Alcohol consumption (Ref. Drinking)**	0.47	0.09	26.08	0.000	1.60	1.34	1.92
**Physical exercise (Ref. Having exercise)**	0.21	0.07	9.44	0.002	1.24	1.08	1.42
**Living expense (Ref. Sufficient)**	0.19	0.08	5.10	0.024	1.21	1.03	1.42
**Sleep time (Ref. <7 hours)**							
7–9 hours	-0.26	0.07	15.21	0.000	0.77	0.68	0.88
≥10 hours	-0.23	0.08	7.99	0.005	0.79	0.68	0.93

CI, confidence interval; LL, lower limit; OR, odds ratio; Ref., reference; S.E., standard error; TTFC, time to first morning cigarette after waking; UL, upper limit.

eTable 8. Subgroup analyses on the relationship between the time interval between the first cigarette smoking in the morning and waking up and the risk of chronic respiratory diseases stratified by sex

	Male							Female						
	B	S.E.	Wald	P	OR	95% CI		B	S.E.	Wald	P	OR	95% CI	
						LL	UL						LL	UL
**TTFC (Ref. No smoking)**														
≤30 minutes	0.57	0.10	30.77	0.000	1.77	1.45	2.17	1.12	0.20	33.09	0.000	3.06	2.09	4.48
>30 minutes	0.49	0.10	24.27	0.000	1.63	1.34	1.97	0.60	0.17	12.96	0.000	1.82	1.31	2.53
**Age, years (Ref. 52–69 years old)**														
70–89	0.49	0.10	23.53	0.000	1.63	1.34	1.98	0.33	0.12	7.80	0.005	1.39	1.10	1.75
≥90	0.31	0.10	8.73	0.003	1.36	1.11	1.66	0.18	0.11	2.82	0.093	1.20	0.97	1.48
**Rurality (Ref. Urban residents)**	-0.34	0.09	14.05	0.000	0.72	0.60	0.85	-0.50	0.10	27.31	0.000	0.61	0.51	0.73
**Alcohol consumption (Ref. Drinking)**	0.45	0.10	18.85	0.000	1.56	1.28	1.91	0.56	0.22	6.64	0.010	1.74	1.14	2.66
**Air cleaning in household (Ref. Having air cleaning)**	—	—	—	—	—	—	—	-0.33	0.15	5.10	0.024	0.72	0.54	0.96
**Living expense (Ref. Sufficient)**	—	—	—	—	—	—	—	0.44	0.11	14.93	0.000	1.55	1.24	1.94
**Physical exercise (Ref. Having exercise)**	0.31	0.09	12.01	0.001	1.37	1.15	1.63	—	—	—	—	—	—	—
**Sleep time (Ref. <7 hours)**														
7–9 hours	-0.29	0.09	10.09	0.001	0.75	0.62	0.89	-0.22	0.10	5.12	0.024	0.80	0.66	0.97
≥10 hours	-0.19	0.11	3.02	0.083	0.82	0.66	1.03	-0.27	0.12	4.87	0.027	0.77	0.61	0.97

BMI, body mass index; CI, confidence interval; LL, lower limit; OR, odds ratio; Ref., reference; S.E., standard error; TTFC, time to first morning cigarette after waking; UL, upper limit; —, not applicable.

Note: Adjusted sex, age, hukou, ventilation in kitchen, distance to the nearest medical institution, physical examination, alcohol consumption, physical exercise, air cleaning in household, lives expense, BMI, sleep time, years of schooling, marital status, fuel of cooking, distance to the arterial traffic, smoking status of people living together, years of smoking.

eTable 9. Subgroup analyses on the relationship between the time interval between the first cigarette smoking in the morning and waking up and the risk of chronic respiratory diseases stratified by age

	52–69 years old							70–89 years old							≥90 years old						
	B	S.E.	Wald	P	OR	95% CI		B	S.E.	Wald	P	OR	95% CI		B	S.E.	Wald	P	OR	95% CI	
						LL	UL						LL	UL						LL	UL
**TTFC (Ref. No smoking)**																					
≤30 minutes	0.91	0.14	43.99	0.000	2.49	1.90	3.26	0.57	0.16	12.73	0.000	1.77	1.29	2.42	0.65	0.16	17.69	0.000	1.92	1.42	2.60
>30 minutes	0.81	0.14	35.55	0.000	2.24	1.72	2.92	0.52	0.15	12.61	0.000	1.69	1.26	2.25	0.36	0.14	6.68	0.010	1.43	1.09	1.87
**Sex (Ref. Male)**	—	—	—	—	—	—	—	-0.37	0.13	8.44	0.004	0.69	0.54	0.89	-0.38	0.11	12.31	0.000	0.69	0.55	0.85
**Rurality (Ref. Urban residents)**	—	—	—	—	—	—	—	—	—	—	—	—	—	—	-0.75	0.10	56.82	0.000	0.47	0.39	0.57
**Distance to medical institutions (Ref. <1.0 km)**																					
1.0–2.9 km	-0.49	0.16	10.06	0.002	0.61	0.45	0.83	—	—	—	—	—	—	—	—	—	—	—	—	—	—
3.0–4.9 km	-0.35	0.20	2.99	0.084	0.70	0.47	1.05	—	—	—	—	—	—	—	—	—	—	—	—	—	—
≥5.0 km	-0.32	0.20	2.61	0.107	0.73	0.50	1.07	—	—	—	—	—	—	—	—	—	—	—	—	—	—
**Alcohol consumption (Ref. Drinking)**	0.54	0.15	13.42	0.000	1.72	1.29	2.29	0.41	0.17	6.12	0.013	1.50	1.09	2.08	0.45	0.17	6.93	0.008	1.56	1.12	2.18
**Physical exercise (Ref. Having exercise)**	—	—	—	—	—	—	—	0.26	0.12	4.65	0.031	1.29	1.02	1.63	—	—	—	—	—	—	—
**Air cleaning in household (Ref. Having air cleaning)**	—	—	—	—	—	—	—	-0.47	0.20	5.77	0.016	0.62	0.42	0.92	—	—	—	—	—	—	—
**BMI (Ref. < 18.5 [Underweight])**																					
18.5–23.9 (Normal weight)	-0.40	0.19	4.66	0.031	0.67	0.47	0.96	—	—	—	—	—	—	—	—	—	—	—	—	—	—
≥24.0 (Overweight/Obesity)	-0.47	0.19	6.09	0.014	0.63	0.43	0.91	—	—	—	—	—	—	—	—	—	—	—	—	—	—
**Sleep time (Ref. <7 hours)**																					
7–9 hours	-0.32	0.11	7.66	0.006	0.73	0.58	0.91	—	—	—	—	—	—	—	-0.36	0.12	10.01	0.002	0.70	0.56	0.87
≥10 hours	-0.14	0.18	0.62	0.431	0.87	0.62	1.23	—	—	—	—	—	—	—	-0.39	0.12	10.68	0.001	0.68	0.54	0.86

BMI, body mass index; CI, confidence interval; LL, lower limit; OR, odds ratio; Ref., reference; S.E., standard error; TTFC, time to first morning cigarette after waking; UL, upper limit; —, not applicable.

Note: Adjusted sex, age, hukou, ventilation in kitchen, distance to the nearest medical institution, physical examination, alcohol consumption, physical exercise, air cleaning in household, lives expense, BMI, sleep time, years of schooling, marital status, fuel of cooking, distance to the arterial traffic, smoking status of people living together, years of smoking.

eTable 10. Subgroup analyses on the relationship between the time interval between the first cigarette smoking in the morning and waking up and the risk of chronic respiratory diseases stratified by rurality

	Registered urban residents							Registered rural residents						
	B	S.E.	Wald	P	OR	95% CI		B	S.E.	Wald	P	OR	95% CI	
						LL	UL						LL	UL
**TTFC (Ref. No smoking)**														
≤30 minutes	0.96	0.15	39.59	0.000	2.62	1.94	3.54	0.60	0.11	29.25	0.000	1.81	1.46	2.25
>30 minutes	0.50	0.14	12.76	0.000	1.64	1.25	2.15	0.60	0.10	34.09	0.000	1.82	1.49	2.23
**Sex (Ref. Male)**	—	—	—	—	—	—	—	-0.34	0.09	14.69	0.000	0.71	0.60	0.85
**Age, years (Ref. 52–69 years old)**														
70–89	0.41	0.14	8.30	0.004	1.51	1.14	2.00	0.42	0.09	21.29	0.000	1.52	1.27	1.81
≥90	0.54	0.14	15.70	0.000	1.71	1.31	2.23	0.09	0.09	1.05	0.305	1.10	0.92	1.31
**Distance to medical institutions (Ref. <1.0 km)**														
1.0–2.9 km	-0.37	0.14	7.25	0.007	0.69	0.53	0.91	-0.05	0.14	0.15	0.698	0.95	0.73	1.24
3.0–4.9 km	-0.68	0.23	8.59	0.003	0.51	0.32	0.80	0.05	0.16	0.10	0.756	1.05	0.77	1.43
≥5.0 km	-0.67	0.26	6.71	0.010	0.51	0.31	0.85	0.26	0.15	3.07	0.080	1.30	0.97	1.75
**Alcohol consumption (Ref. Drinking)**	0.41	0.17	5.57	0.018	1.51	1.07	2.12	0.47	0.11	18.72	0.000	1.60	1.30	1.99
**Physical exercise (Ref. Having exercise)**	0.59	0.12	25.37	0.000	1.80	1.43	2.27							
**Living expense (Ref. Sufficient)**	—	—	—	—	—	—	—	0.19	0.09	4.45	0.035	1.21	1.01	1.45
**Sleep time (Ref. <7 hours)**														
7–9 hours	—	—	—	—	—	—	—	-0.26	0.08	10.35	0.001	0.77	0.66	0.90
≥10 hours	—	—	—	—	—	—	—	-0.29	0.10	8.61	0.003	0.75	0.62	0.91

BMI, body mass index; CI, confidence interval; LL, lower limit; OR, odds ratio; Ref., reference; S.E., standard error; TTFC, time to first morning cigarette after waking; UL, upper limit; —, not applicable.

Note: Adjusted sex, age, hukou, ventilation in kitchen, distance to the nearest medical institution, physical examination, alcohol consumption, physical exercise, air cleaning in household, lives expense, BMI, sleep time, years of schooling, marital status, fuel of cooking, distance to the arterial traffic, smoking status of people living together, years of smoking.

eTable 11. Subgroup analyses on the relationship between the time interval between the first cigarette smoking in the morning and waking up and the risk of chronic respiratory diseases stratified by smoking status

	No smoking (Ref.)	Ex-smoker							Current smoker						
		B	S.E.	Wald	P	OR	95% CI		B	S.E.	Wald	P	OR	95% CI	
							LL	UL						LL	UL
**TTFC (Ref. No smoking)**															
≤30 minutes	1.000	0.93	0.12	65.98	0.000	2.54	2.03	3.19	0.39	0.12	10.39	0.001	1.47	1.16	1.86
>30 minutes	1.000	0.67	0.10	43.70	0.000	1.95	1.60	2.37	0.29	0.12	5.92	0.015	1.33	1.06	1.68
**Sex (Ref. Male)**	1.000	-0.35	0.08	20.80	0.000	0.71	0.61	0.82	-0.32	0.08	17.30	0.000	0.73	0.62	0.84
**Age, years (Ref. 52–69 years old)**															
70–89	1.000	0.40	0.09	20.99	0.000	1.49	1.25	1.76	0.45	0.09	26.98	0.000	1.57	1.33	1.87
≥90	1.000	0.23	0.08	7.42	0.006	1.26	1.07	1.48	0.31	0.08	13.40	0.000	1.36	1.15	1.61
**Rurality (Ref. Urban residents)**	1.000	-0.49	0.07	44.94	0.000	0.62	0.53	0.71	-0.38	0.08	25.52	0.000	0.68	0.59	0.79
**Distance to medical institutions (Ref. <1.0 km)**															
1.0–2.9 km	1.000	-0.16	0.11	2.36	0.125	0.85	0.69	1.05	-0.33	0.10	9.96	0.002	0.72	0.59	0.88
3.0–4.9 km	1.000	-0.26	0.14	3.61	0.057	0.77	0.59	1.01	-0.26	0.13	3.86	0.049	0.77	0.59	1.00
≥5.0 km	1.000	0.06	0.13	0.20	0.655	1.06	0.82	1.37	-0.08	0.13	0.38	0.540	0.92	0.72	1.19
**Alcohol consumption (Ref. Drinking)**	1.000	0.47	0.12	15.27	0.000	1.60	1.26	2.02	0.34	0.11	9.46	0.002	1.40	1.13	1.74
**Physical exercise (Ref. Having exercise)**	1.000	0.29	0.08	13.74	0.000	1.34	1.15	1.56	—	—	—	—	—	—	—
**Living expense (Ref. Sufficient)**	1.000	—	—	—	—	—	—	—	0.27	0.09	8.53	0.003	1.31	1.09	1.56
**Sleep time (Ref. <7 hours)**															
7–9 hours	1.000	-0.23	0.07	9.81	0.002	0.79	0.69	0.92	-0.28	0.08	13.31	0.000	0.76	0.65	0.88
≥10 hours	1.000	-0.18	0.09	4.02	0.045	0.84	0.70	1.00	-0.24	0.09	6.74	0.009	0.79	0.65	0.94

BMI, body mass index; CI, confidence interval; LL, lower limit; OR, odds ratio; Ref., reference; S.E., standard error; TTFC, time to first morning cigarette after waking; UL, upper limit; —, not applicable.

Note: Adjusted sex, age, hukou, ventilation in kitchen, distance to the nearest medical institution, physical examination, alcohol consumption, physical exercise, air cleaning in household, lives expense, BMI, sleep time, years of schooling, marital status, fuel of cooking, distance to the arterial traffic, smoking status of people living together, years of smoking.

eTable 12. Subgroup analyses on the relationship between the time interval between the first cigarette smoking in the morning and waking up and the risk of chronic respiratory diseases stratified by smoking consumption

	1–10 cigarettes per day							>10 cigarettes per day						
	B	S.E.	Wald	P	OR	95% CI		B	S.E.	Wald	P	OR	95% CI	
						LL	UL						LL	UL
**TTFC (Ref. No smoking)**														
≤30 minutes	0.57	0.13	19.28	0.000	1.77	1.37	2.28	0.73	0.11	45.23	0.000	2.08	1.68	2.57
>30 minutes	0.40	0.10	17.06	0.000	1.50	1.24	1.81	0.72	0.12	34.25	0.000	2.05	1.61	2.60
**Sex (Ref. Male)**	-0.31	0.08	17.55	0.000	0.73	0.63	0.85	-0.34	0.08	18.98	0.000	0.71	0.61	0.83
**Age, years (Ref. 52–69 years old)**														
70–89	0.54	0.09	38.57	0.000	1.72	1.45	2.04	0.36	0.09	17.52	0.000	1.44	1.21	1.70
≥90	0.36	0.08	17.92	0.000	1.43	1.21	1.69	0.24	0.08	8.27	0.004	1.27	1.08	1.50
**Rurality (Ref. Urban residents)**	-0.40	0.07	29.97	0.000	0.67	0.58	0.77	-0.48	0.08	40.46	0.000	0.62	0.53	0.72
**Distance to medical institutions (Ref. <1.0 km)**														
1.0–2.9 km	-0.28	0.10	7.29	0.007	0.76	0.62	0.93	-0.19	0.11	3.35	0.067	0.82	0.67	1.01
3.0–4.9 km	-0.37	0.14	7.47	0.006	0.69	0.53	0.90	-0.14	0.14	1.08	0.299	0.87	0.67	1.13
≥5.0 km	-0.06	0.13	0.24	0.621	0.94	0.73	1.21	0.05	0.13	0.14	0.709	1.05	0.81	1.35
**Alcohol consumption (Ref. Drinking)**	0.35	0.11	9.58	0.002	1.41	1.14	1.76	0.55	0.12	22.72	0.000	1.73	1.38	2.17
**Physical exercise (Ref. Having exercise)**	—	—	—	—	—	—	—	0.24	0.08	9.49	0.002	1.28	1.09	1.49
**Air cleaning in household (Ref. Having air cleaning)**	—	—	—	—	—	—	—	-0.25	0.11	5.01	0.025	0.78	0.63	0.97
**Living expense (Ref. Sufficient)**	0.22	0.09	5.73	0.017	1.25	1.04	1.50	0.19	0.09	4.12	0.042	1.21	1.01	1.45
**Sleep time (Ref. <7 hours)**			14.06	0.001										
7–9 hours	-0.25	0.08	11.17	0.001	0.78	0.67	0.90	-0.26	0.08	11.97	0.001	0.77	0.67	0.89
≥10 hours	-0.27	0.09	8.33	0.004	0.77	0.64	0.92	-0.15	0.09	2.83	0.093	0.86	0.72	1.03

BMI, body mass index; CI, confidence interval; LL, lower limit; OR, odds ratio; Ref., reference; S.E., standard error; TTFC, time to first morning cigarette after waking; UL, upper limit; —, not applicable.

Note: Adjusted sex, age, hukou, ventilation in kitchen, distance to the nearest medical institution, physical examination, alcohol consumption, physical exercise, air cleaning in household, lives expense, BMI, sleep time, years of schooling, marital status, fuel of cooking, distance to the arterial traffic, smoking status of people living together, years of smoking.
